# Six-fold director field configuration in amyloid nematic and cholesteric phases

**DOI:** 10.1038/s41598-019-48996-3

**Published:** 2019-09-02

**Authors:** Massimo Bagnani, Paride Azzari, Salvatore Assenza, Raffaele Mezzenga

**Affiliations:** 1 ETH Zurich, Department of Health Sciences and Technology, Schmelzbergstrasse 9, LFO E23, Zurich, 8092 Switzerland; 2 ETH Zurich, Department of Materials, Wolfgang-Pauli-Strasse 10, Zurich, 8093 Switzerland; 3 Present Address: Departamento de Física Teórica de la Materia Condensada, Universidad Autónoma de Madrid, Madrid, Spain

**Keywords:** Liquid crystals, Biomaterials - proteins

## Abstract

Chiral liquid crystals, or cholesteric phases, have been widely studied in the last decades, leading to fundamental advances and a multitude of applications and technologies. In general, the rich phenomenology of these systems depends directly on the molecular traits and conditions of the system, imposing precise symmetry to the resulting nematic field. By selecting amyloid fibrils as model filamentous chiral colloids, we report an unprecedented breadth of liquid crystalline morphologies, where up to six distinct configurations of the nematic field are observed under identical conditions. Amyloid-rich droplets show homogeneous, bipolar, radial, uniaxial chiral and radial chiral nematic fields, with additional parabolic focal conics in bulk. Variational and scaling theories allow rationalizing the experimental evidence as a subtle interplay between surface and bulk energies. Our experimental and theoretical findings deepen the understanding of chiral liquid crystals under confinement, opening to a more comprehensive exploitation of these systems in related functional materials.

## Introduction

Liquid crystals are a class of materials that combine the long-range order typical of solids with the fluidity of liquids. The morphology of liquid crystal colloids (or tactoids) has been in the research focus during the last decades because of their remarkable spontaneous self-organization into a rich variety of configurations with distinct physical properties^1–3^. Particularly, elucidating the mechanisms behind the self-organization of cholesteric liquid crystals in constrained environments with deformable interfaces is key for creating programmable bio-inspired materials^4^.

The emerging structures and associated functionalities result from an intriguing interplay between bulk elasticity, surface anchoring and intrinsic chirality of the mesogens, dictating the equilibrium configuration of the tactoid^5,6^. Due to this subtle balance of the energy terms, the shape and director field configuration of the tactoid are very sensitive to various chemical and physical factors, such as temperature, chiral and achiral additives, external fields and confinement^7–9^. Confinement-induced reorganization of anisotropic particles in spherical regions is of particular interest, due to the possibility of experimentally generating droplets with controlled curvature for fundamental studies of particle packing, self-assembly, and relaxation of colloidal solutions^10–14^.

Tactoids have been shown to display multiple configurations, including homogeneous and bipolar^15^, and cholesteric droplets^6–14^ and artificially induced structural transitions in nematic and cholesteric droplets have been subject of multiple studies^3,16,17^. Yet, a single system capable of attaining all these morphologies at once, with spontaneous transitions, has been experimentally elusive. Recent studies have reported the continuous transformation of tactoids from homogeneous to bipolar in carbon nanotube suspensions^15^ and only recently the bipolar to uniaxial cholesteric transition for amyloid fibril suspensions^6^. In particular, it was found that destabilized dispersions of beta-lactoglobulin amyloid fibrils positioned within the Isotropic-Nematic biphasic region nucleate from an unstable isotropic phase into droplets displaying three different morphologies (homogeneous, bipolar, uniaxial cholesteric), depending on the volume of the tactoid. These findings were rationalized by means of scaling arguments, which correctly predicted the occurrence of the three configurations at increasing volumes^6^, making that chiral colloidal system the one with the richest lyotropic behaviour.

In the present work, we show that by allowing amyloid tactoids to grow further in size, sediment and phase separate macroscopically from the feeding isotropic continuous phase, this system may exhibit an even richer palette of morphologies. We demonstrate that as many as six different symmetries of the nematic field are present, i.e. the double of those previously observed. Apart from the three cases reported by Nystrom *et al*.^6^, the aggregates also show droplets with: (i) radial nematic symmetry; (ii) radial cholesteric (reminiscent of onion-like morphologies) and (iii) macroscopic bulk phases characterized by parabolic focal conics with discrete orientation of the nematic field. Beside the two order-order transitions already known, i.e. the homogeneous-bipolar and bipolar-uniaxial cholesteric transitions^6^, we report and characterize a third and additional transition that amyloid tactoids spontaneously undergo in order to minimize the free energy, from *uniaxial cholesteric* to *radial cholesteric* (radial Frank–Pryce architecture^18^). To the best of our knowledge, this is the first reported case of spontaneous uniaxial to radial cholesteric transition occurring in colloidal systems, which contrasts with previous approaches actively modifying the anchoring energy by means of electric fields^19^, changes in temperature^20^, surfactants and microfluidic devices^14,21^. Scaling arguments are employed to elucidate the physical nature of the first two transitions and the occurrence of internal defects in the radial nematic and chiral nematic droplets, but fail in capturing the essence of the third newly observed transition. Therefore, we introduce a model for the nematic field based on the Frank-Oseen energy, which captures the field distribution of *all* the experimentally observed configurations in the droplets and their corresponding transition volumes, including the newly observed *uniaxial cholesteric* to *radial cholesteric transition* and we discuss the physical origin of the sixth and last parabolic focal conics morphology, where the nematic field becomes discontinuous, making an analogy to the classical Kelvin space filling problem.

## Results and Discussion

### Amyloid tactoid phases

**Figure 1:**
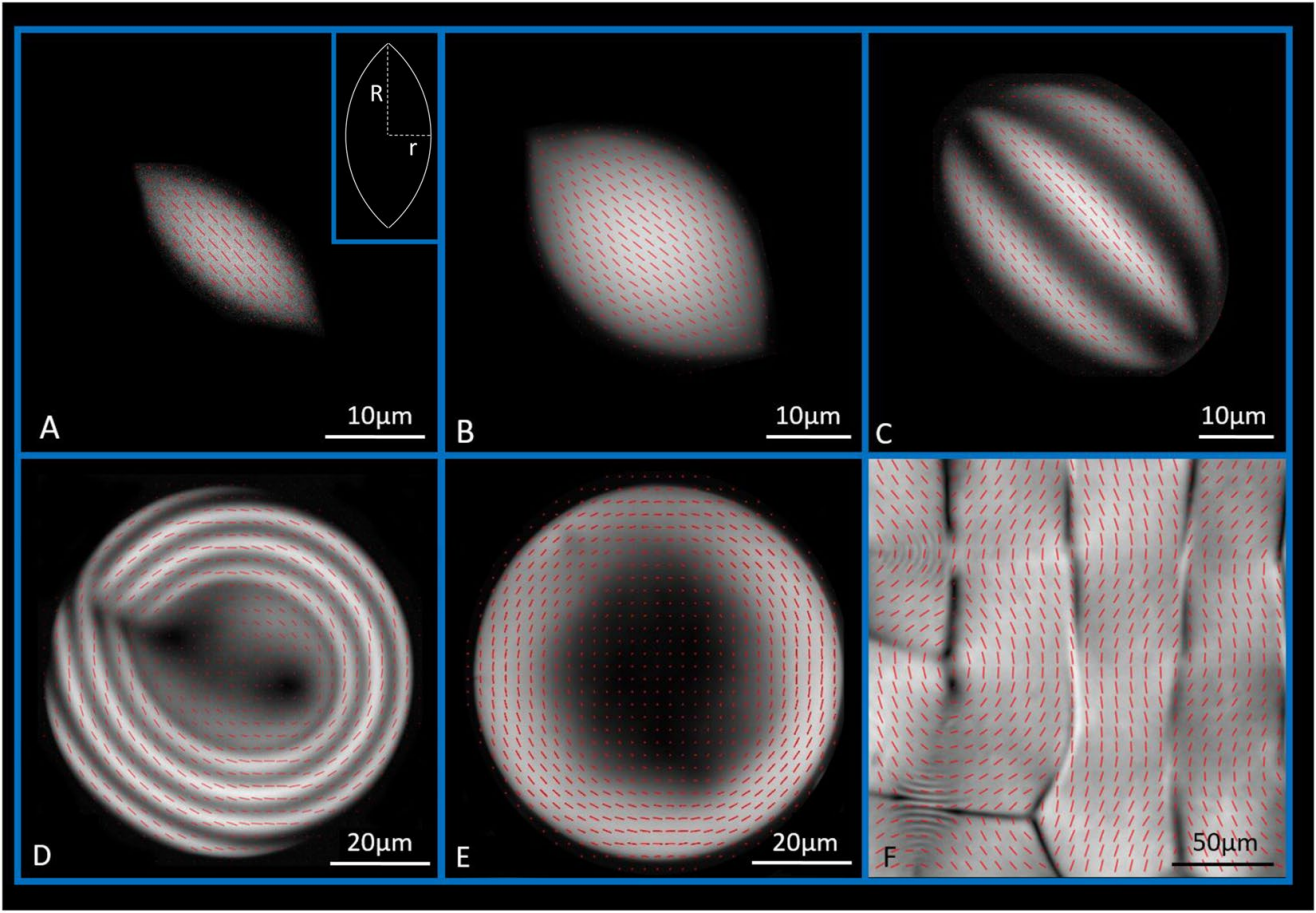
Director field, obtained with LC Polscope, for homogeneous (**A**), bipolar (**B**), uniaxial cholesteric (**C**), radial cholesteric (**D**), radial nematic (**E**) e bulk phase (**F**). The caption in (**A**) shows R and r, the major and minor axes of the tactoid.

Aqueous dispersions of amyloid fibrils show an isotropic-nematic phase transition that is well described by Onsager theory^22^. This is followed by a nucleation and growth mechanism leading to the formation of five different classes of tactoids, until macroscopic phase separation is reached (Fig. S1) through coalescence and sedimentation. The droplets differ on their shape and nematic director field configuration, which varies depending on their confinement. Microscopic analysis allows measuring their major (*R*) and minor (*r*) axes (see caption in Fig. 1A) and therefore their aspect ratio (*α* = *R*/*r*) and volume (*V* ~ *r*^2^*R*). For cholesteric droplets, the periodicity (or pitch) is easily measurable as twice the band to band distance. Additionally, an LC PolScope device enables discrimination of the different classes of tactoids (see Fig. 1).

At growing volumes, droplets morphologies appear according to the sequence homogeneous nematic, bipolar nematic, uniaxial cholesteric and radial nematic and cholesteric. These transitions are induced by the spontaneous and continuous increase in droplet volume during equilibration. The first and smallest tactoids to nucleate are the homogeneous, characterized by the highest aspect ratio values (highly elongated droplets) and by a director field that is always parallel to the long axis of the tactoid (Fig. 1A). The second droplet class is characterized by smaller aspect ratios and a bipolar director field, that follows the interface of the droplet (Fig. 1B). Once bipolar tactoids reach a critical volume, the uniaxial cholesteric configuration is preferred and the typical striped pattern appears (Fig. 1C). Further increase in volume pushes a third transition from uniaxial to radial cholesteric droplets, with cholesteric rings disposed radially around the center of the droplet (Fig. 1D). A metastable form of this radial organization (radial nematic, Fig. 1E) is discussed later in the text. These tactoids are characterized by a spherical shape, thus showing aspect ratio values very close to one.

The free-energy landscape associated with the first three classes of amyloid droplets and which enables capturing their appearance at increasing droplet volume in the right order, has been recently analysed by Nystrom *et al*. via scaling of the Frank-Oseen theory, taking the functional form^6^:

1 $$F\sim \frac{{K}_{1,3}{r}^{2}}{R}+\gamma Rr[1+\omega {(\frac{r}{R})}^{2}]+{K}_{2}{(q-{q}_{\infty })}^{2}{r}^{2}R$$

The first term is referred to as bending energy, where *K*_1,3_ is the Frank elastic constant for splay and bending (here assumed identical). The second term is the surface energy, where *γ* is the isotropic surface tension and *ω* the anchoring strength. Lastly, the last term gives the twisting energy, where *K*_2_ is the Frank elastic constant for twist, *q* the average twist and *q*_∞_ = 2*π*/*p*_∞_ the chiral wave number, where *p*_∞_ is the natural pitch of the system (see Supplementary Information for further details on the Frank-Oseen functional). One important feature of the free energy in Equation (1), is the different dependence of the various terms on the volume. In particular, the bending energy scales as ${V}^{\frac{1}{3}}$, the surface energy as ${V}^{\frac{2}{3}}$, while the twist energy as V. Therefore, at increasing volumes, the three terms contribute differently. The homogeneous-to-bipolar and bipolar-to-uniaxial cholesteric transitions were shown to take place at the critical values $\frac{V}{\alpha }\sim {(\frac{{K}_{1,3}}{\omega \gamma {\alpha }^{2}})}^{3}$ and $\frac{V}{\alpha }\sim {(\frac{\gamma \omega }{{\alpha }^{2}{q}_{\infty }^{2}{K}_{2}})}^{3}$, respectively. Nonetheless, no indication of additional transitions at larger volumes were provided, neither experimentally nor theoretically, since as we show below, equation (1) cannot predict any additional transition at higher volumes, calling for refined theoretical formalisms. In the following sections, the spontaneous uniaxial-to-radial cholesteric transition, reported here for the first time, is analysed in detail both experimentally and theoretically.

### Uniaxial to radial cholesteric spontaneous transition

One of the most interesting phenomena analysed in this paper is the spontaneous transition of the droplets from uniaxial cholesteric into a radial cholesteric configuration, causing the orientation of the amyloids on the droplet surface to switch from (imperfect) homeotropic to tangential alignment. This transition was first demonstrated by Volovik and Lavrentovich in systems based on liquid crystal esters (nonylhydrobenzoic-acid). Nevertheless, as mentioned above, this change of alignment was achieved tuning the surface tension by increasing temperature^23^ or adding surfactant molecules^24^. To the best of our knowledge, amyloid based liquid crystals are the first system showing this transition spontaneously.

**Figure 2:**
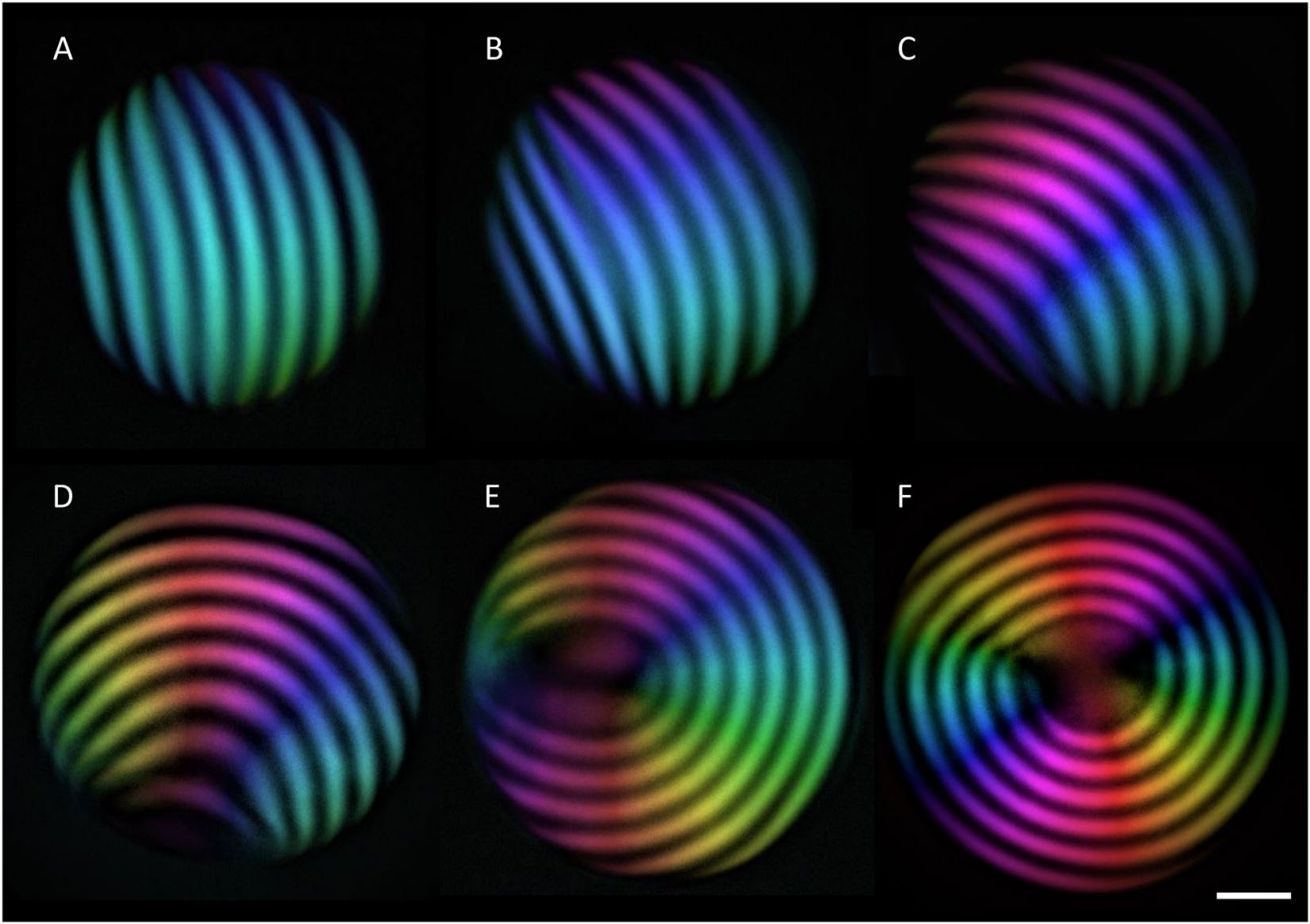
Snapshots of the uniaxial to radial cholesteric transition analysed by LC PolScope imaging: Upon increasing droplet volume, the uniaxial cholesteric configuration of the director field (**A**) becomes energetically too expensive, and the cholesteric bands start gradually to bend (**B**–**D**) favouring planar anchoring until the onion-like radial cholesteric configuration is reached (**E** and **F**). The scale bar applies to all images and is equal to 15 µm.

In the amyloid case, characterized by large anchoring strength^25^, tangential alignment is preferred. Hence, in the uniaxial cholesteric configuration, the homeotropic alignment of the mesogens at the interface is realized at a large anchoring cost. At a critical droplet volume, the periodic bands start to bend and rearrange into an onion-like structure consisting of multiple concentric spherical shells with radially oriented helical axes. During this transition, the rods progressively align along the interface, until the radial cholesteric configuration is achieved, as shown in Fig. 2. As explained below, this transition is led by the anchoring energy, which becomes more important at increasing volumes of the tactoid, and scales as ∼rR. The increasing stress originating from the anchoring energy of growing droplets in the uniaxial cholesteric configuration can be relaxed by reintroducing bending, which scales as ∼r^2^/R and is thus less expensive at large volumes (see equation (1)).

### Defect configurations in spherical confinement

Defects in the director field are common in liquid crystals^26,27^. In the case of spherical confinement, the principal reason for the formation of topological defects is the induced tangential alignment. When a nematic director field is constrained to be tangent to a spherical boundary, it is unavoidably forced to have topological defects^26^. A previous study^14^ based on fluorinated oil emulsions of cellulose nanocrystals, which imposes parallel anchoring due to enormous interfacial energies, showed that the morphology of the droplets and their defects are strongly influenced by the confinement, which was controlled by a micro-fluidic device and via the concentration of nanoparticles added in solution. Different classes of defects in spherical droplets were also associated with a change in DNA concentration and osmotic stress in DNA based cholesteric droplets^28,29^.

**Figure 3:**
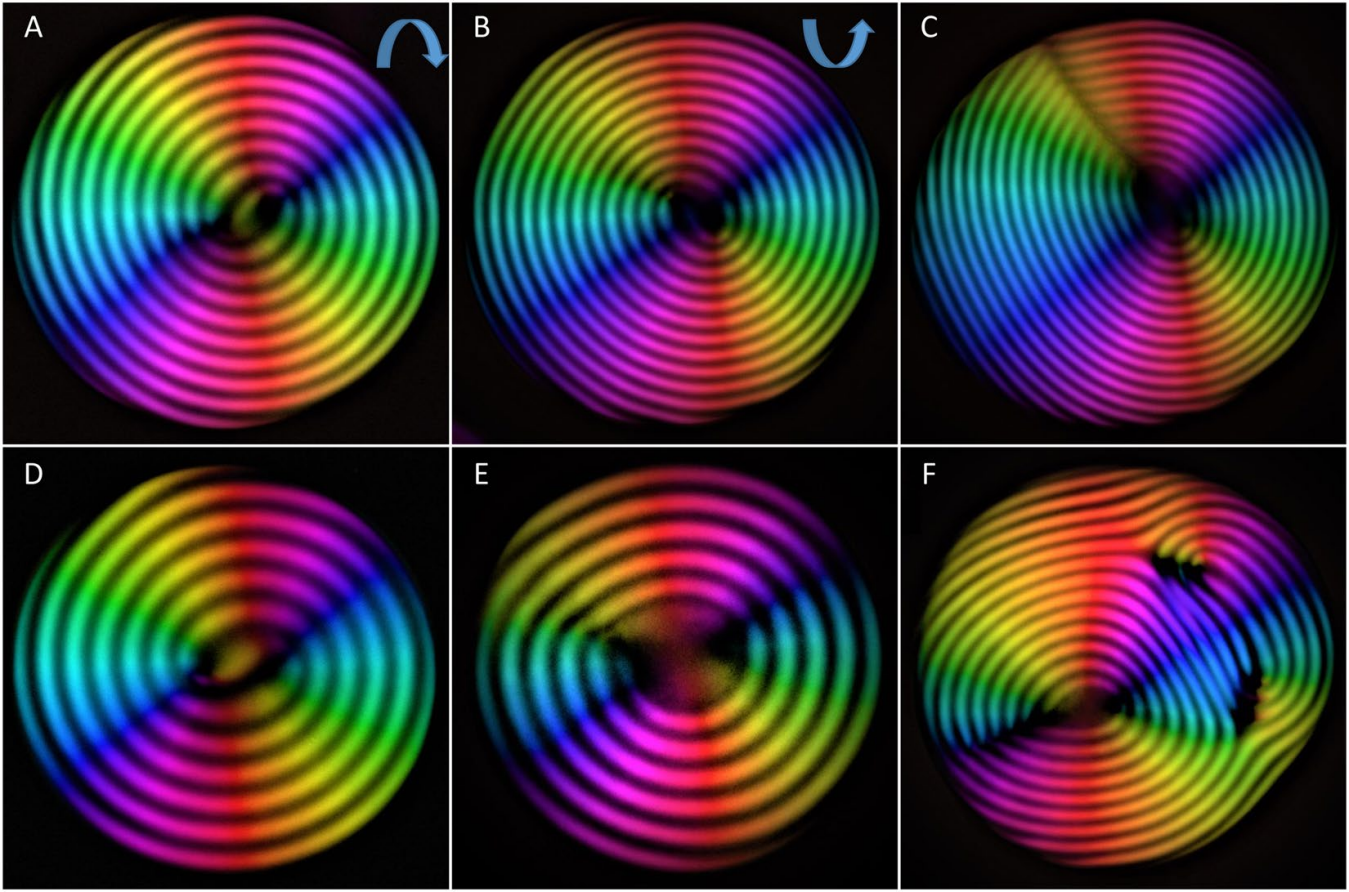
Different classes of defects in radial cholesteric droplets: (**A**) droplet showing a double spiralized pattern rotating clockwise. (**B**) Droplet showing a double spiralized pattern rotating anti-clockwise. (**C**) Droplet showing concentric layers with a radial disclination line. (**D**) Droplet with continuous spiral pattern (punctual defects). (**E**) Droplet with concentric layers with a nematic core at the center of the droplet. (**F**) Droplet showing different defects after merging with a second tactoid. The cholesteric pitch in the droplet is equal to 15 µm for each of the droplets shown in figure.

Due to the microscopic scale of the nematic liquid crystal, the topological defects are easily observable under an optical microscope, using birefringence imaging analysis. Four classes of defects characterize the equilibrated structures of amyloid-based radial cholesteric droplets. Figure 3A,B show a double spiral structure with a dislocation located in the center of the droplet associated with a ${\lambda }^{\frac{1}{2}}$ disclination and a dislocation^30^. When these spirals are visualized under a cross-polarized microscope, the twist appears to turn clockwise or anti-clockwise, depending on the viewing plane (see Fig. S2). Hence, the spirals are not chiral and are not related to the cholesteric handedness of amyloid fibrils^6^. A radial disclination line (Fig. 3C) running through the droplet radius is often observed. In some instances, the droplets show a continuous spiral pattern (association of two ${\lambda }^{\frac{1}{2}}$ disclinations^31^, see Fig. 3D). In the vast majority of cases, however, the fingerprint pattern is lost in the center of the droplets, where a core is formed (Fig. 3E). We note that birefringence of the liquid crystal is maintained in the droplet core (Fig. 3E), suggesting that the center is a nematic phase, unlike the system reported by^32^. The size of the core appears to be independent of the droplet volumes and roughly equal to the cholesteric pitch (see Supplementary Information for a more detailed discussion).

Additional defects (Fig. 3F), such as dislocations associated with partial insertion or removal of the concentric layers, have larger energetic costs and therefore are typically expelled following equilibration. Below a more detailed discussion about defects in the bulk phase is reported.

### Scaling theory

**Figure 4:**
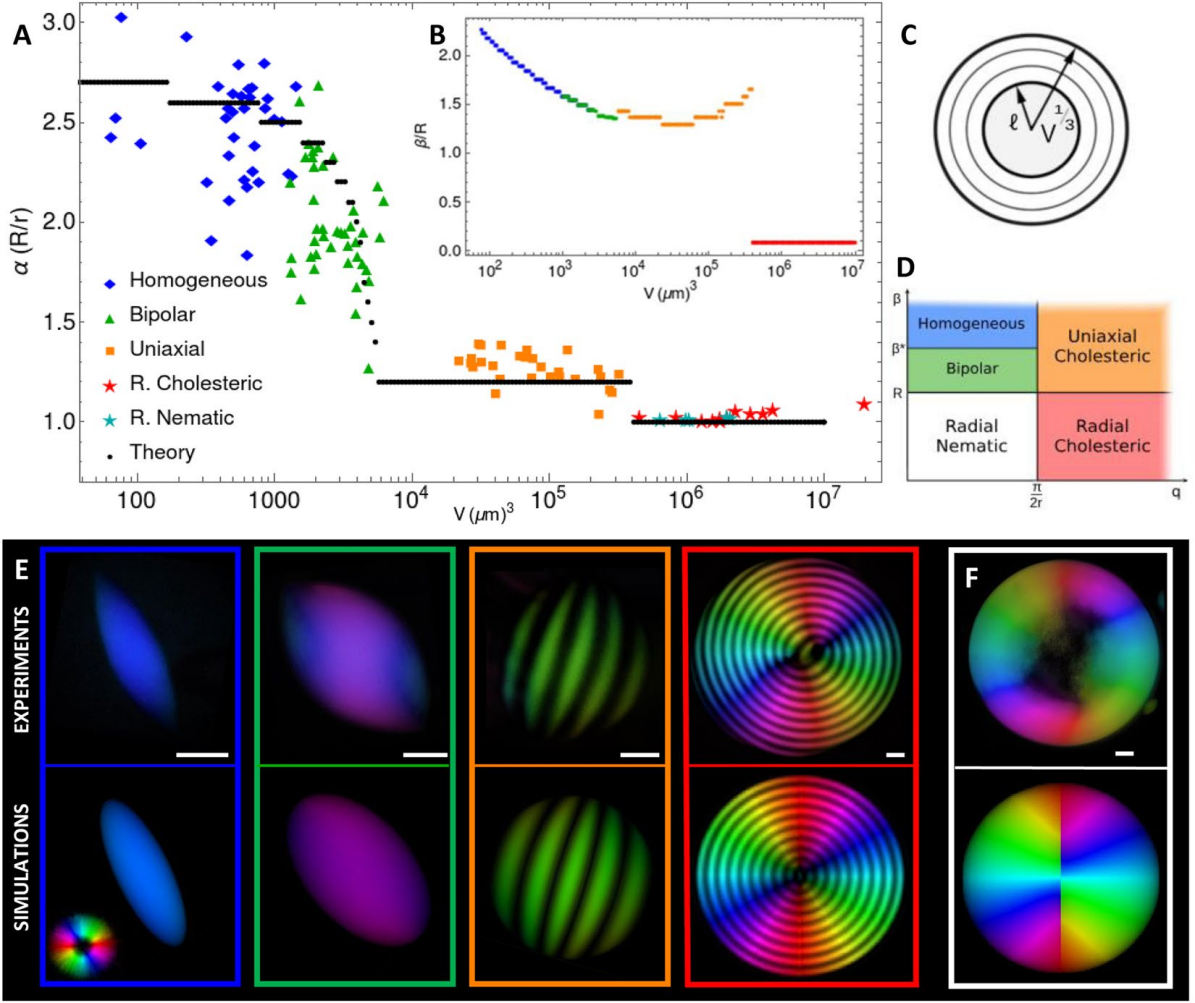
Comparison of the results of the theoretical model with experimental data. (**A**) Droplet aspect ratio vs volume. (**B**) Dependence of ratio β/R on volume. (**C**) Schematic of the radial cholesteric droplet within the scaling theory. (**D**) Classification of droplets within the nematic field employed in the variational theory. (**E**) Comparison between experimental and theoretical tactoid morphologies: First row from left to right: LC PolScope images of homogeneous, bipolar, uniaxial cholesteric and radial cholesteric droplets. Second row: corresponding theoretical predictions visualized via PolScope simulation, obtained by minimizing the free energy. (**F**) Experimental and theoretical tactoid morphologies for the radial nematic configuration, not predicted by the minimization. The scale bars correspond to 10 µm.

The uniaxial to radial cholesteric transition can be rationalized by recognizing that at large volumes the free energy of a droplet is dominated by the surface term (see Supplementary Information). Hence, the tactoid will display a spherical shape (surface tension) with a tangential configuration of the nematic field (anchoring). Nevertheless, this configuration cannot encompass the whole droplet, as this would lead to extremely large bulk energies in the center. Specifically, in the central part of the tactoid the bending caused by the radial cholesteric configuration is energetically more expensive than untwisting, which is thus preferred by the system. (see Supplementary Information). Therefore, the tactoid can be divided into two parts (see Fig. 4C): a nematic inner core, of radius $\,\ell$, with energy given by the untwisted nucleus ${F}_{core}\sim {K}_{2}\,{\ell }^{3}{q}_{\infty }^{2}$, and an outer cholesteric shell, with elastic energy dominated by the bend term ${F}_{shell}\sim {K}_{3}({V}^{1/3}-\ell )$, see Supplementary Information for the derivation. The total energy is given by the sum of the two, $F={F}_{core}+{F}_{shell}$ and therefore by minimizing *F* in respect to $\ell$, we get an equilibrium value of the core radius ${\ell }^{\ast }=\sqrt{\frac{{K}_{3}}{{K}_{2}}}\frac{1}{{q}_{\infty }}$, independent of volume as observed in experiments (see Supplementary Information for further discussion). Unfortunately, while the scaling is rather successful in predicting the presence of a defect in the core of the radial cholesteric droplets, it fails in predicting the location of the *uniaxial-to-radial cholesteric* transition (see Supplementary Information). Indeed, the transition is driven by the same mechanisms that lead the *homogeneous-to-bipolar* transition of the simple nematic droplets (namely bend and surface terms in Eq. (1)). A scaling theory based on these ingredients fails to tell apart the two transitions, which are predicted to take place at the same volume up to numerical prefactors. Hence, a more robust and complex analysis of the free energy landscape becomes necessary as discussed below.

### Variational theory

To rationalize the entire breadth of the experimental findings, we developed a theory based on the Frank-Oseen free energy^33^ (see Supplementary Information). The success of this theory in reproducing faithfully the distribution of the nematic field in the four major classes of tactoids, the correct transition volumes, and the accurate aspect ratios is highlighted in Fig. 4.

Ideally, one should minimize the total free energy with respect to the director field *n* and the shape of the droplet at constant volume *V*^33^. This prohibitive task is simplified considering a nematic field of the form $n=\,\cos (q\beta \sigma +u){e}_{u}-\,\sin (q\beta \sigma +u){e}_{\sigma }$, using oblate spheroidal coordinates $(\sigma ,\tau ,u)$, dependent on the parameters *β*, *q*, as discussed in detail in the Supplementary Information. The nematic field represents a helix with wave number *q*, whose axis is given by the coordinate lines of *σ*. Its great advantage is that it recapitulates the five different configurations observed experimentally, which correspond to specific values of the parameters. Indeed, *q* gives information on whether the tactoid is in the cholesteric or nematic regime, while *β* continuously tunes between the various configurations (Fig. 4D). More in detail, the cholesteric cases are obtained for *qr* > *π*/2, with uniaxial and radial tactoids corresponding to $\beta \gtrsim R$ and $\beta \lesssim R$, respectively. The nematic configurations are found for *qr* $<$ *π*/2, with the bipolar droplets corresponding to *R* $<$ $\beta \lesssim {\beta }^{\ast }\equiv 1.5R$, and the homogeneous ones obtained for $\beta \gtrsim {\beta }^{\ast }$.

Interestingly, besides the four main classes of tactoids observed shown in Fig. 4C, a fifth configuration of the nematic field can also be predicted by the variational theory and can be represented in correspondence of low *q* and *β* (white region in Fig. 4D). Nevertheless, such combination of parameters never minimizes the free energy; accordingly, experimentally these radial nematic droplets are seldom observed (Fig. 4F), inferring their metastable nature over the more stable chiral nematic radial droplets: by Eq. (1), their difference in energy can be indeed estimated to be of the order of ~${K}_{2}{{q}_{\infty }}^{2}{V}_{shell}$, where *V*_*shell*_ is the volume of the outer shell.

Assuming the droplet to be a prolate ellipsoid of aspect ratio *α*, the Frank-Oseen energy corresponding to this nematic field was minimized numerically by tuning the parameters *q*, *β* and *α* (see Supplementary Information). The elastic constants and surface tension were slightly adjusted from^6^ to better fit the data (see Supplementary Information). Moreover, at each volume the experimental value of the pitch was implemented in the chiral term of the energy (see Supplementary Information).

The results are shown in Fig. 4. The theory correctly predicts the order of appearance of the various configurations and the monotonic decay of the aspect ratio with growing volume. Moreover, it captures well the transition volumes, when experimental values of the constants are used. In Fig. 4E (bottom row) representative tactoids obtained from the variational theory have been rendered using Jones matrices^34^ to simulate the LC PolScope (see Supplementary Information). Homogenous and bipolar tactoids display a larger aspect ratio, with a parameter *β* continuously decreasing and larger than the major axis of the droplet. The transition value ${\beta }^{\ast }=1.5R$ from homogenous to bipolar has been chosen by visual analysis of the Jones matrix simulations. As the volume increases, the aspect ratio drops to a smaller value, with *β* still greater than *R* and increasing, indicating droplets in the uniaxial cholesteric regime (the volume is now large enough for the condition *qr* $>$ *π*/2 to be fulfilled). Finally, the transition to radial cholesteric tactoids at even larger volumes is shown clearly by the ratio *β*/*R*, which discontinuously jumps to almost zero (Fig. 4B), denoting a radial director field. The simultaneous jump of the aspect ratio to *α* = 1 further shows that the shape is a sphere. A more in-depth discussion on the pitch behaviour is included in the Supplementary Information. A non-monotonic behaviour of *β*/*R* is predicted, which is the result of a subtle interplay between twist and bend energies with the superficial anchoring.

### Bulk-phase topology

**Figure 5:**
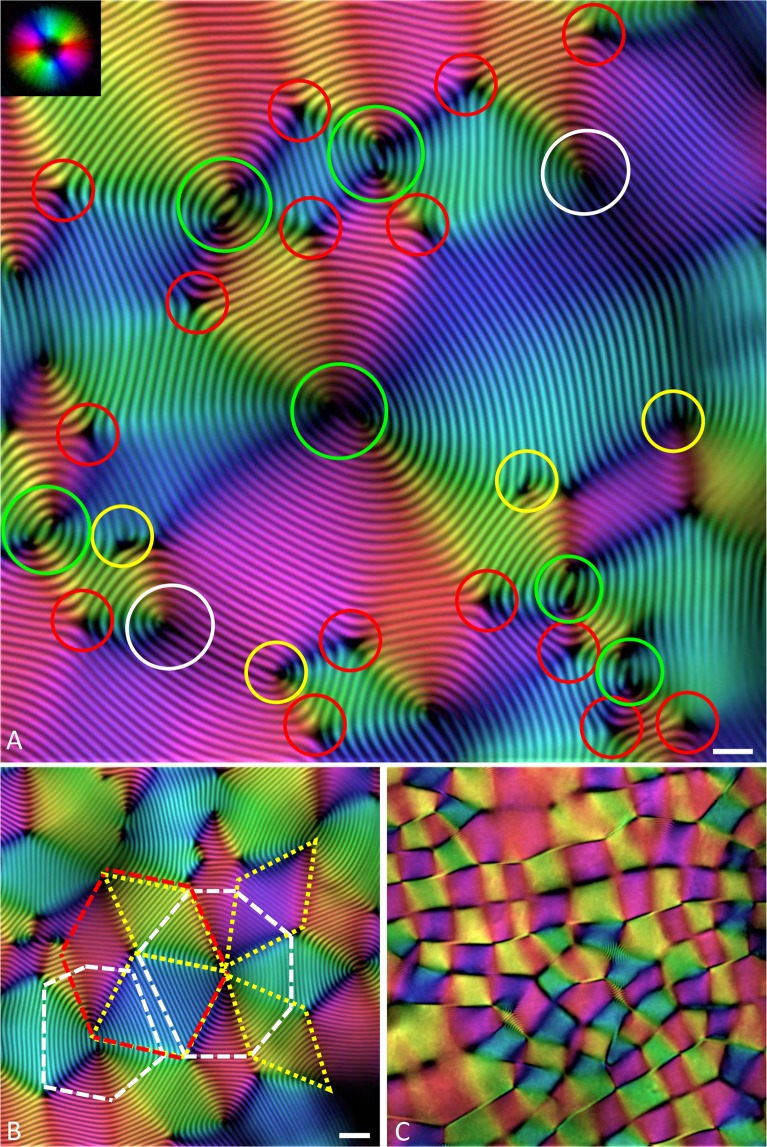
Cholesteric bulk phase: (**A**) Defects in cholesteric: yellow circles correspond to dislocations (bifurcating lines), red circles to ${{\boldsymbol{\lambda }}}^{-\frac{1}{2}}$ disclinations, white circles to ${{\boldsymbol{\lambda }}}^{\frac{1}{2}}$disclinations and green ones to double spiral patterns formed by the association of two ${{\boldsymbol{\lambda }}}^{\frac{1}{2}}$ disclinations. The scale bar is equal to 30 µm (**B**) hexagonal pattern and (**C**) parabolic focal conic pattern. The scale bar applies to the all the images and is equal to 50 µm.

When stored vertically for several weeks, the different classes of birefringent crystalline droplets eventually coalesce into a macroscopic aggregate (see Fig. S3 in Supplementary Information). This phase separation is achieved through very slow sedimentation, thanks to the difference in amyloid concentration between the isotropic and anisotropic phases. After joining the bulk cholesteric phase, the cholesteric droplets merge trapping numerous defects. In particular, Fig. 5A shows the different classes of disclinations and dislocations observed in the bulk cholesteric phase, highlighted in different colours. The most frequently observed disclinations are triple point patterns (${{\rm{\lambda }}}^{-\frac{1}{2}}$ disclinations), formed by the contact point of three radial cholesteric droplets, followed by double spiral patterns (association of two ${{\rm{\lambda }}}^{\frac{1}{2}}$ disclinations), often present in the core of the droplets, and finally a few cases of singlet ${{\rm{\lambda }}}^{\frac{1}{2}}$ disclinations^30,31^. Only rare examples of dislocation defects are observed in the form of bifurcations, since they are typically expelled during equilibration.

During bulk formation, the spherical droplets deform their shape leading to the formation of a faceted pattern (Fig. 5B), which allows a more efficient packing of the spherical domains. Indeed, assimilating the radial cholesteric tactoids as soft spherical objects with the tendency of surface minimization, the optimal packing coincides with the solution of the old Kelvin problem of minimum surface area per bubble^35^, which leads to the formation of a polyhedral honeycomb structure which are at the origin of the structure first described by Bouligand^36^.

Further equilibration leads to the emergence of domains arranged onto a square lattice with a period of 40 µm and separated by defect lines, where the nematic director field changes discontinuously (Figs 5C, S4 and S5). A small helical pitch of 5 µm (three times smaller than in the previous configurations) is visible in a few domains, while it vanishes in most cases. These features are compatible with either a polygonal texture, where lamellar sheets span the space between the glass plates alternating upwards and downwards directions in a zigzag fashion^37^, or a parabolic focal conic texture, characterized by an approximately squared lattice defined by a regular array of interlocking parabolic line defects^38^. Rotation of the typical dark crosses upon rotating the sample between cross polarizers indicates that the latter structure is the one being actually observed^30–34^ (see Supplementary Video 1), which was further confirmed by the alternating blue and orange coloring of domains when a full-wave retardation plate was considered^34^ (Fig. S4). The parabolic focal conic structure has been previously observed and analysed in systems based on DNA, cellulose, oligomers or lipids^31,38–41^, as well as in nature. E.g. in the metallic beetle Chrysina gloriosa^42^, where it originates the characteristic green iridescent colour, but its origin in the present system as a result of deformation of droplets prior/during coalescence has not been discussed before.

To summarize, we have studied the evolution of amyloid-based liquid crystalline droplets, unveiling a system with unprecedented polymorphism. Five different kinds of tactoids were observed and characterized at increasing volumes, and rationalization of the experimental results was achieved via a combination of scaling arguments and a variational theoretical model, which are in both qualitative and quantitative agreement with data. In particular, our findings show for the first time a spontaneous *uniaxial-to-radial cholesteric transition* for sufficiently large volumes. Lastly, a sixth configuration with discontinuous orientation of the nematic field was observed during the equilibration of the cholesteric bulk phase, showing a parabolic focal conic pattern with discrete and discontinuous orientation of the nematic field. To the best of our knowledge, this is the first system exhibiting spontaneously all the above-mentioned configurations, and a theoretical comprehensive model, which was still missing, is developed capable to describe such a complete and complex phenomenology of single tactoids.

These findings offer a new playground for further investigating the behaviour of this new class of chiral liquid crystals. From a broader perspective, the generality of the theory introduced here suggests that similar features could be observed in other cholesteric systems, and sets a versatile toolbox to develop optimized protocols for the production of chiral tactoids with targeted properties.

## Materials and Methods

### Samples preparation

β-lactoglobulin monomers were extracted and purified from whey protein following the protocol described by Vigolo *et al*.^43^. To produce amyloid fibrils, 6 grams of β-lactoglobulin monomers were dissolved into 300 mL of pH 2 milli Q and the dispersion incubated at 90 °C for 5 hours. The solution was mixed during all the heating process using a magnetic bar at 150 rpm to avoid fibrils gelation at the interface. To stop the fibrillization process, the solution was immediately quenched by immersion of the flask into an ice-water mixture. In order to produce short β-lactoglobulin amyloid fibrils, the preformed native fibrils were stirred using a magnetic bar at 1200 rpm for one week, as discussed in Bagnani *et al*.^25^. The solution was dialyzed against 10 L pH 2 Milli-Q water (pH adjusted adding 1 M HCl) using a semipermeable membrane (Spectra/Por dialysis membrane 1, MWCO 100 kDa) for 4 days with daily bath change. The solution was then up concentrated with reverse osmosis (using semipermeable membrane Spectra/Por dialysis membrane 1, MWCO 6-8 kDa) against a 5 wt% PEG (MW 35 kDa) pH 2 solution.

### Atomic force microscopy characterization

At the end of the stirring process, a small aliquot of the sample was taken for AFM analysis. The solution was diluted into a final concentration of 0.01 wt% in pH 2 Milli-Q water. 20 μL of the resulting solution were deposited and incubated for 2 min onto freshly cleaved mica and then rinsed with Milli-Q water, and dried with compressed air flow. AFM experiments were performed using a Multimode VIII scanning probe microscope (Bruker, USA) and images were acquired in tapping mode at ambient conditions. The average contour lengths of amyloid fibrils and their contour length distributions^25^ were obtained analysing the AFM images with the open source code FiberApp^44^ (see SI).

### Optical microscopy

In order to analyse the liquid crystalline structures under polarized microscope, 40 uL of solution were injected using a pipette into glass cuvettes (0.2 × 4 × 40 mm^3^, VitroTubes, Vitrocom). The filled cuvettes sealed with epoxy were stored vertically at room temperature for a few days (up to few months) and examined with optical microscope after equilibration. The optical analysis was realized combining a cross-polarized microscope and a LC-PolScope universal compensator, enabling to examine the orientation of the amyloids forming the structures evolving in the isotropic-nematic coexisting phase, as previously discussed^25^. The volumes were estimated using ImageJ measuring the length of the two axes of the tactoids. For the nematic tactoids, the volume was estimated as $1.2{r}^{2}R$, taking into account also the tip angle, while for the cholesteric ones as $\frac{4}{3}\pi {r}^{2}R$.

## Supplementary information

Supplementary information

Rotation of Parabolic Focal Conic defects

## References

[1] Kim Y-K, Shiyanovskii SV, Lavrentovich OD (2013). Morphogenesis of defects and tactoids during isotropic-nematic phase transition in self-assembled lyotropic chromonic liquid crystals. J. Phys. Condens. Matter.

[2] Bouligand Y (2008). Liquid crystals and biological morphogenesis: Ancient and new questions. Comptes Rendus Chim..

[3] Zhou Ye, Bukusoglu Emre, Martínez-González José A., Rahimi Mohammad, Roberts Tyler F., Zhang Rui, Wang Xiaoguang, Abbott Nicholas L., de Pablo Juan J. (2016). Structural Transitions in Cholesteric Liquid Crystal Droplets. ACS Nano.

[4] Seeman NC, Belcher AM (2002). Emulating biology: Building nanostructures from the bottom up. Proc. Natl. Acad. Sci..

[5] Prinsen P, van der Schoot P (2003). Shape and director-field transformation of tactoids. Phys. Rev. E.

[6] Nyström G, Arcari M, Mezzenga R (2018). Confinement-induced liquid crystalline transitions in amyloid fibril cholesteric tactoids. Nat. Nanotechnol..

[7] Peng C, Lavrentovich OD (2015). Chirality Amplification and Detection by Tactoids of Lyotropic Chromonic Liquid Crystals. Soft Matter.

[8] Banks T, Casher A (1980). Chiral symmetry breaking in confining theories. Nucl. Physics, Sect. B.

[9] Nayani Karthik, Fu Jinxin, Chang Rui, Park Jung Ok, Srinivasarao Mohan (2017). Using chiral tactoids as optical probes to study the aggregation behavior of chromonics. Proceedings of the National Academy of Sciences.

[10] Manoharan VN, Elsesser MT, Pine DJ (2003). Dense Packing and Symmetry in Small Clusters of Microspheres. Science (80-.)..

[11] Teich EG, van Anders G, Klotsa D, Dshemuchadse J, Glotzer SC (2016). Clusters of polyhedra in spherical confinement. Proc. Natl. Acad. Sci..

[12] De Nijs B (2015). Entropy-driven formation of large icosahedral colloidal clusters by spherical confinement. Nat. Mater..

[13] Zhang B, Cheng X (2016). Structures and Dynamics of Glass-Forming Colloidal Liquids under Spherical Confinement. Phys. Rev. Lett..

[14] Li Y (2016). Colloidal cholesteric liquid crystal in spherical confinement. Nat. Commun..

[15] Jamali V (2015). Experimental realization of crossover in shape and director field of nematic tactoids. Phys. Rev. E - Stat. Nonlinear, Soft Matter Phys..

[16] Bajc J, Zumer S (1997). Structural transition in chiral nematic liquid crystal droplets in an electric field. Phys. Rev. E.

[17] Bajc J, Bezic J, Zumer S (1995). Chiral nematic droplets with tangential anchoring and negative dielectric anisotropy in an electric field. Phys. Rev. E.

[18] Parker, R. M. *et al*. The Self-Assembly of Cellulose Nanocrystals: Hierarchical Design of Visual Appearance. *Adv*. *Mater*. **30** (2018). 10.1002/adma.201704477 29250832

[19] Kitzerow HS (1994). Polymer-dispersed liquid crystals From the nematic curvilinear aligned phase to ferroelectric films. Liq. Cryst..

[20] Kurik MV, Lavrentovich D (1982). Negative-positive monopole transitions in cholesteric liquid crystals. Eksp. Teor. Fiz.

[21] Wang P-XX, Hamad WY, MacLachlan MJ (2016). Polymer and Mesoporous Silica Microspheres with Chiral Nematic Order from Cellulose Nanocrystals. Angew. Chemie - Int. Ed..

[22] Jung JM, Mezzenga R (2010). Liquid crystalline phase behavior of protein fibers in water: Experiments versus theory. Langmuir.

[23] Volovik GE, Lavrentovich OD (1983). Topological dynamics of defects: boojums in nematic drops. Sov. Phys. JETP.

[24] Tran L (2017). Change in stripes for cholesteric shells via anchoring in moderation. Phys. Rev. X.

[25] Bagnani Massimo, Nyström Gustav, De Michele Cristiano, Mezzenga Raffaele (2018). Amyloid Fibrils Length Controls Shape and Structure of Nematic and Cholesteric Tactoids. ACS Nano.

[26] Lopez-Leon T, Fernandez-Nieves A (2011). Drops and shells of liquid crystal. Colloid Polym. Sci..

[27] Lavrentovich, O. D. Defects and Textures in Liquid Crystals. *Handb*. *Liq*. *Cryst*. 189–241, 10.1142/9789812778581_0011 (2014).

[28] Leonard, M., Hong, H., Easwar, N. & Strey, H. H. Soft matter under osmotic stress. **42**, 5823–5827 (2001).

[29] Stanley CB, Hong H, Strey HH (2005). DNA cholesteric pitch as a function of density and ionic strength. Biophys. J..

[30] Seč D, Porenta T, Ravnik M, Žumer S (2012). Geometrical frustration of chiral ordering in cholesteric droplets. Soft Matter.

[31] Leforestier A, Livolant F (1993). Supramolecular ordering of DNA in the cholesteric liquid crystalline phase: an ultrastructural study. Biophys. J..

[32] Schopohl N, Sluckin TJ (1987). Defect Core Structure in Nematic Liquid Crystals. Phys. Rev. Lett..

[33] Virga Epifanio G. (1994). Variational Theories for Liquid Crystals.

[34] Lien A (1990). Extended Jones matrix representation for the twisted nematic liquid‐crystal display at oblique incidence. Appl. Phys. Lett..

[35] Weaire, D. *The Kelvin Problem*. (Taylor & Francis, 1997).

[36] Bouligand Y, Livolant F (1984). The organization of cholesteric spherulites. J. Phys..

[37] Shimamura, K. Liquid Crystalline Structure of Aqueous H y droxy prop ylcellulose. **111**, 107–111 (1983).

[38] Donald AM, Viney C, Ritter AP (1986). The parabolic focal conic texture in a lyotropic liquid-crystalline polymer. Liq. Cryst..

[39] Roman M, Gray DG (2005). Parabolic focal conics in self-assembled solid films of cellulose nanocrystals. Langmuir.

[40] Sinitsyna OV, Bobrovsky AY, Meshkov GB, Yaminsky IV, Shibaev VP (2017). Direct Observation of Changes in Focal Conic Domains of Cholesteric Films Induced by Ultraviolet Irradiation. J. Phys. Chem. B.

[41] Meister R, Hallé MA, Dumoulin H, Pieranski P (1996). Structure of the cholesteric focal conic domains at the free surface. Phys. Rev. E - Stat. Physics, Plasmas, Fluids, Relat. Interdiscip. Top..

[42] Sharma V, Crne M, Park JO, Srinivasarao M (2009). Structural Origin of Circularly Polarized Iridescence in Jeweled Beetles. Science (80-.)..

[43] Vigolo D (2017). Continuous Isotropic-Nematic Transition in Amyloid Fibril Suspensions Driven by Thermophoresis. Sci. Rep..

[44] Usov I, Mezzenga R (2015). FiberApp: An open-source software for tracking and analyzing polymers, filaments, biomacromolecules, and fibrous objects. Macromolecules.

