# Supplementary material for: Six-fold director field configuration in amyloid nematic and cholesteric phases
Source: Sci Rep. 2019 Sep 2;9:12654. doi: 10.1038/s41598-019-48996-3 (PMC6718687; doi:10.1038/s41598-019-48996-3)
Supplement: Supplementary file 1 — Supplementary information [file 41598_2019_48996_MOESM1_ESM.docx]

Supplementary Information for

**Six-fold director field configuration in amyloid nematic and cholesteric phases**

Massimo Bagnani^1^†, Paride Azzari^1^†, Salvatore Assenza^1^ and Raffaele Mezzenga^1,2^*

^1^ETH Zurich, Department of Health Sciences and Technology, Schmelzbergstrasse 9, LFO E23 Zurich 8092, Switzerland.

^2^ETH Zurich, Department of Materials, Wolfgang-Pauli-Strasse 10, Zurich 8093, Switzerland.

†Both authors contributed equally to this work.

*Correspondence to: [raffaele.mezzenga@hest.ethz.ch](mailto:raffaele.mezzenga@hest.ethz.ch)

**Length distribution and phase diagram**

The protocol used to produce amyloid ﬁbrils, has been described in detail in a previous publication^1^. In brief, 6 grams of β-lactoglobulin monomers were dissolved into 300 mL of pH 2 milli Q and the dispersion incubated at 90 °C for 5 hours while was stirring using a magnetic bar at 150 rpm. A small sample aliquot (1 μL) was taken after 168 hours of stirring at 1200rpm for AFM analysis. The fibril samples were diluted into a ﬁnal concentration of 0.01 wt%, in pH 2 Milli-Q water and then 20 μL solution were deposited onto freshly cleaved mica, incubated for 2 min, rinsed with Milli-Q water, and dried with compressed air ﬂow. AFM experiments were performed using a Multimode VIII scanning probe microscope (Bruker, USA) and images were acquired in tapping mode at ambient conditions. The average contour lengths of amyloid fibrils and their contour length distributions^1^ were obtained
analysing the AFM images with the open source code FiberApp^2^ . The phase diagram was constructed as described in detail in a previous publication^1^, by up concentrating the solution until a fully nematic phase was achieved without crossing the sol/gel transition. Solutions with different concentrations were then prepared with progressive dilution and mixing using pH 2 Milli-Q water (with dilution steps of 0.1 wt%) down to concentrations where the samples were completely isotropic. The absence of birefringence domains was investigated first between cross polarizers and then after equilibration with polarized microscopy^1^.


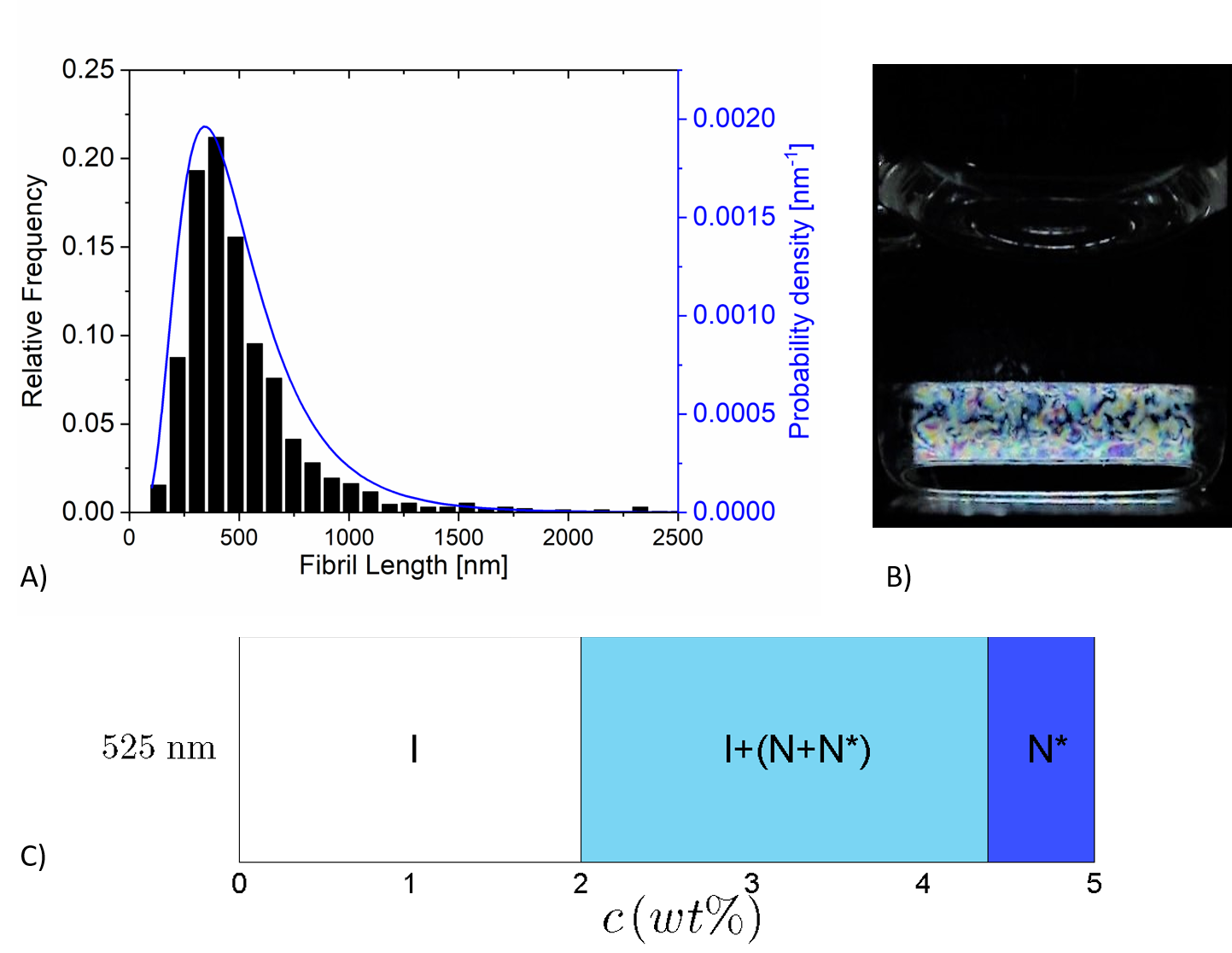


**Figure S1 System characterization.** A) Amyloid length distribution in black, overlapped with a log-normal fit in blue. B) Macroscopic phase separation seen between cross-polarizers. C) Phase diagram at pH2 for the system studied. Adapted from^1^.


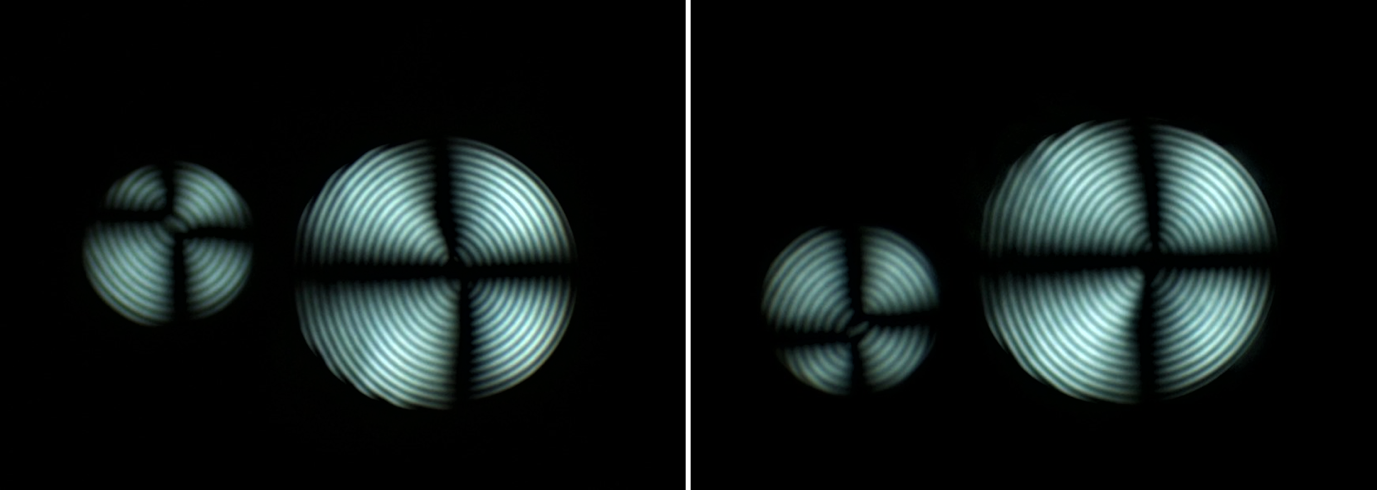


**Figure S2 Spiral pattern visualization.** Two different radial cholesteric droplet visualized from the top (left image) and from the bottom (right). The twist of spiral turns from anticlockwise (left) to clockwise (right) in the big droplet and vice-versa in the small one. In radial cholesteric droplets, the pitch is independent from the volume and equal to 15µm.

**Bulk phase defects characterization**

The different classes of birefringent crystalline droplets achieve a macroscopic phase separation (Fig. S1B) through slow sedimentation, thanks to the difference in amyloid concentration between the isotropic and the anisotropic phases. The bulk phase was analysed using a LC-PolScope universal compensator, to examine the orientation of the fibrils inside the structures formed in the isotropic-nematic coexisting phase. Different classes of defects are highlighted in Fig. S3.


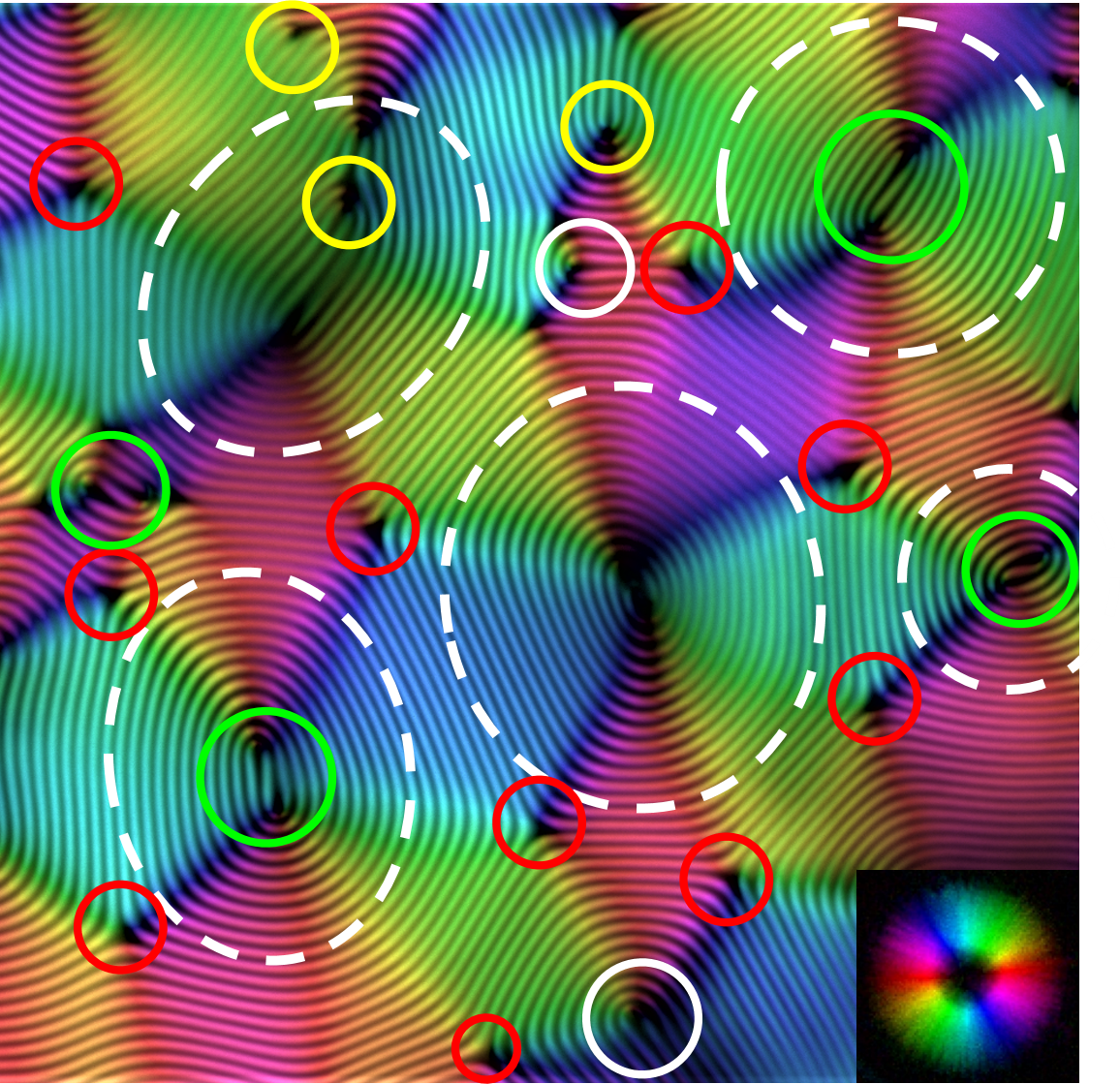


**Figure S3 Bulk phase defects.** Overview LC PolScope image of the different classes of cholesteric bulk phase defects highlighted by circles. White dashed lines highlight the radial droplets merged in the bulk phase. Yellow circles correspond to dislocations, red circles to -π disclinations, white circles to $+\pi$disclinations and green ones to double spiral patterns formed by the association of two +π disclinations ^3,4^. The cholesteric pitch is equal to 15µm.

**Parabolic focal conic bulk phase**

Further equilibration of the bulk phase leads to the formation of squared domains^4–6^ with a grain size of the order of 40 µm (Fig. S4). With a full-wave retardation plate, a blue color appears where the slow axis of the birefringent sample is aligned with the slow axis of the wave plate, and an orange color appears where the fast axis of the sample is aligned with the slow axis of the wave plate ^6^ (Fig. S4).


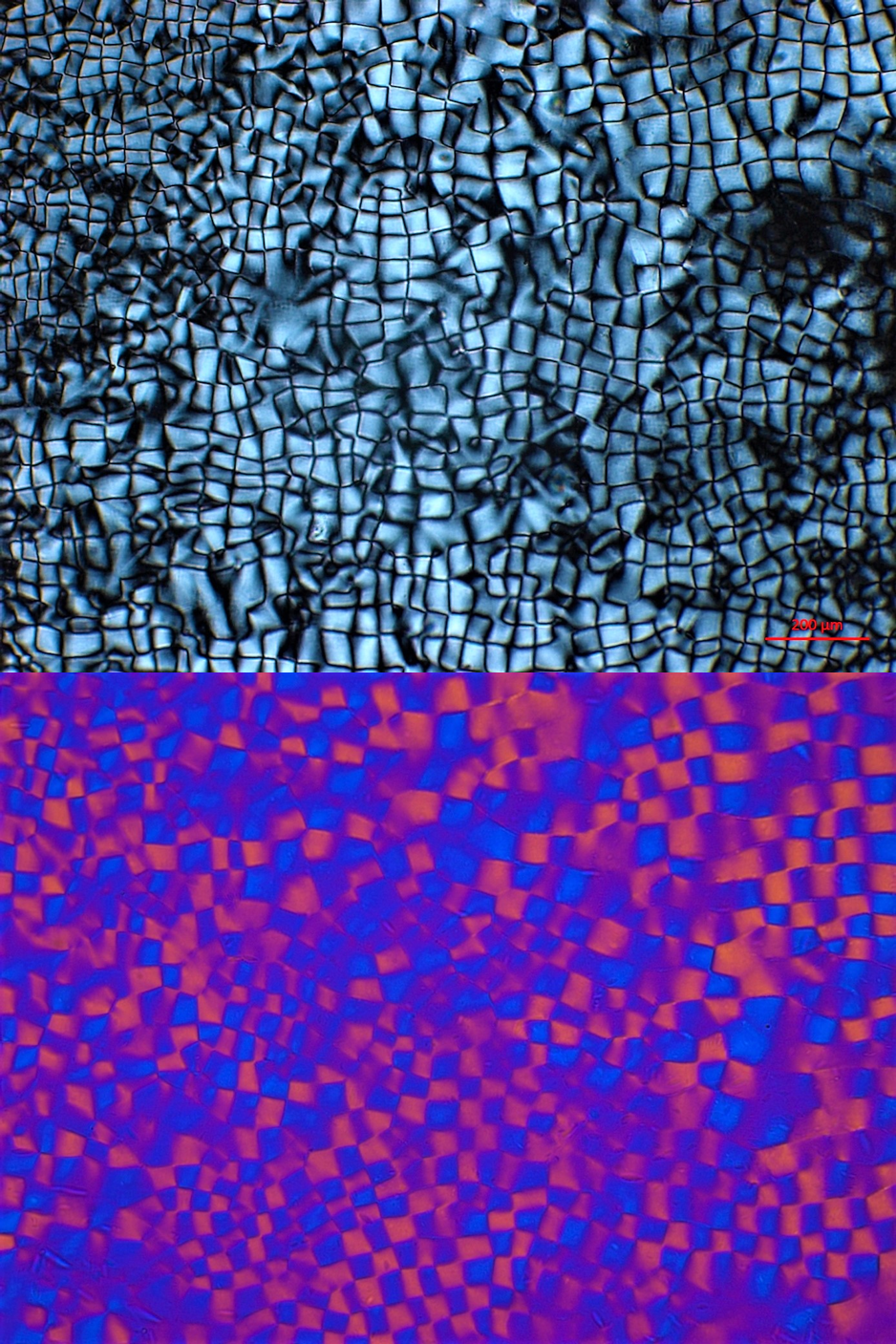


**Figure S4 Parabolic focal conic configuration.** On top cross-polarized microscope image of the parabolic focal conic domains, at the bottom the structure observed with a full wave retardation plate. Scale bar applies to both images.


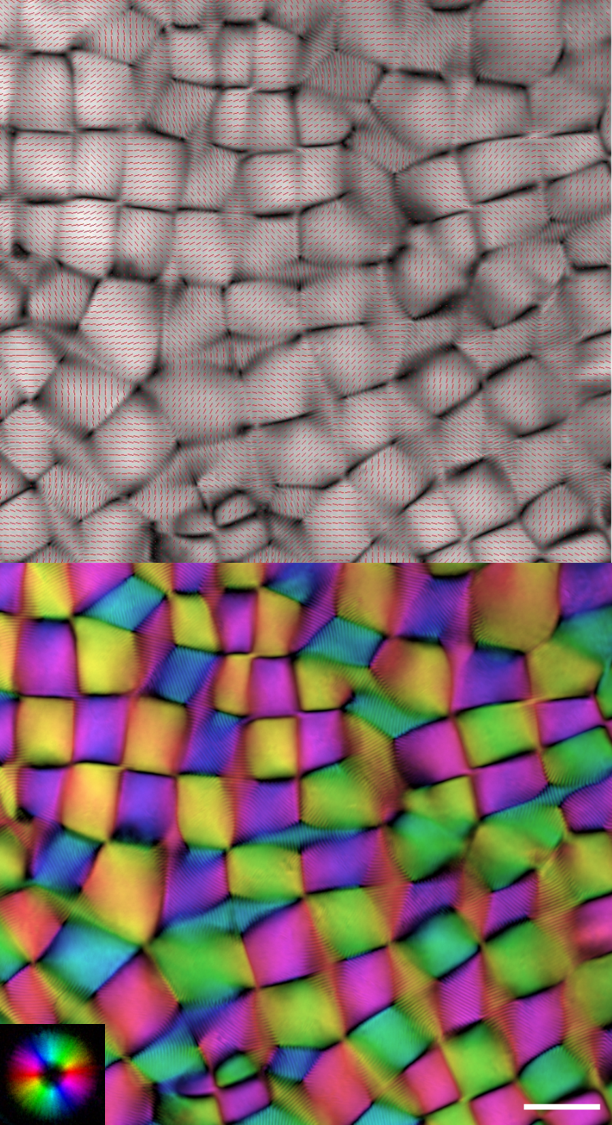


**Figure S5 PolScope images of parabolic focal conic domains.** On the top image the lines represent the director field, represented in colors in the bottom image. The director varies discontinuously from one domain to a neighboring one and the pitch is equal to 5µm. Scale bar is equal to 50 µm.

In this configuration, the pitch value is three times smaller than the one observed in the radial cholesteric tactoids and appears to vanish in most of the domains. The helical pitch of 5 µm is sometimes visible (fig. S5). This smaller pitch, compared to the one that characterizes the newly formed cholesteric bulk phase, suggests an increased packing of the rods.

**Theoretical Background**

We compute the free energy$F$ as the sum of a bulk term $F_{B}$ given by the Frank-Oseen elastic energy^7^ and a superficial term $F_{S}$, given by the anisotropic surface tension^7^.

$$F_{B}=\int_{V} K_{1}\left( \nabla\cdot n \right)^{2}+K_{2}\left( n\cdot\nabla\times n-q_{\infty} \right)^{2}+K_{3}\left( n\times\nabla\times n \right)^{2} dV$$

$$F_{S}=\gamma\int_{S} 1+\omega\left( n\cdot t \right)^{2} dS$$

where $n$ is the nematic field;$t$ is the normal to the surface *S* of the tactoid; $K_{1,2,3}$ are the Frank elastic constants for splay, twist and bend deformations, respectively; $\gamma$the surface tension; $\omega$ the anchoring strength; and $q_{\infty}=2\pi/p_{\infty}$ is the chiral wave number, where $p_{\infty}$ is the natural pitch of the system. Here and in the following we do not consider the effects of the saddle-splay term ^7^.

**Scaling theory.** By making explicit the dependence of $F$ on the volume, one obtains

$$F=F_{B}+F_{S}=V^{1/3}\tilde{F}_{B}+V^{2/3}\tilde{F}_{S}$$

where $\tilde{F}_{B,s}$ are respectively the bulk and surface term calculated at unit volume. At large volumes, the surface energy term dominates over the bulk term and therefore a sphere with tangential alignment will give the minimum free energy.

We consider the radial cholesteric droplet as a sphere divided into two pieces, namely a nematic core of radius $\mathcal{l}$ and a cholesteric outer shell. At fixed volume $V$, the outer shell has radius scaling with $V^{\frac{1}{3}}$, and a thickness of $V^{\frac{1}{3}}-\mathcal{l}$.

Inside the core, the Frank-Oseen energy is equal to the energy of the untwisted cholesteric:

$$F_{core}=K_{2}\int_{core} q_{\infty}^{2}dV\sim K_{2}\mathcal{l}^{3}q_{\infty}^{2};$$

As for the outer shell, the leading term is the bend energy, whose density scales as the square of radius of curvature: $\left( n\times\nabla\times n \right)^{2}\sim\frac{1}{\rho^{2}}$, where $\rho$ is the distance from the center of the droplet.

$$F_{shell}=K_{3}\int_{shell} \frac{1}{\rho^{2}}dV\sim K_{3}\left( V^{\frac{1}{3}}-\mathcal{l} \right);$$

By minimizing $F=F_{core}+F_{shell}=K_{2}\mathcal{l}^{3}q_{\infty}^{2}+K_{3}\left( V^{\frac{1}{3}}-\mathcal{l} \right)$, we get the equilibrium radius as $\mathcal{l}^{*}=\frac{\sqrt{\frac{K_{3}}{K_{2}}}}{q_{\infty}}$ independent of volume. Using the values of the variational approach, we estimate $\mathcal{l}^{*}=4 \mu m$.

We measured experimentally the core of the radial cholesteric droplets $\mathcal{l}$ at various volumes V. The results are shown in Figure S6A. The size of the core fluctuates in a small interval (between 10 and 20 µm) for volumes within a range encompassing almost two orders of magnitude, which is compatible with the prediction of constant$\mathcal{l}$. Figure S6B shows that $\mathcal{l}$ follows a probability distribution centered at 14.2 µm and with standard deviation 2.7 µm, very close to the value of the pitch in the radial cholesteric regime of 15 µm, although somewhat larger than the scaling prediction $\mathcal{l}^{*}$. Such an offset could be expected, as scaling results are always given up to numerical prefactors typically of the order of units.


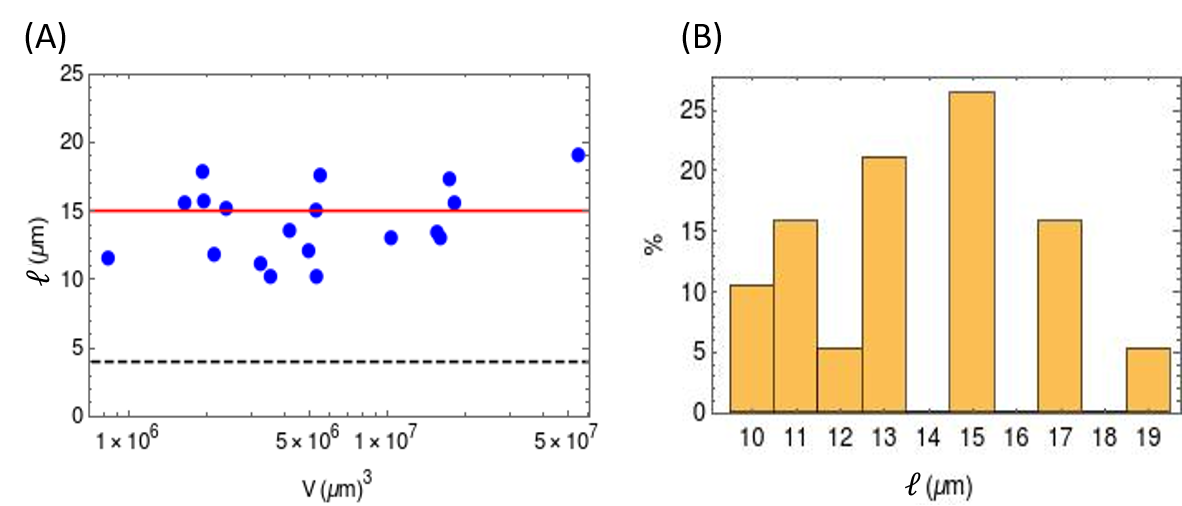


**Figure S6 Core radius in radial cholesteric droplets.** A) Core radius as a function of the volume, the value of the pitch in the radial cholesteric regime 15 µm is highlighted in red while the black dashed line (4 µm) represents the scaling prediction. B) Distribution of the core radius, with mean 14.2 µm and standard deviation 2.7 µm.

By substituting$\mathcal{l}^{*}$ into$F,$ we get the equilibrium energy scaling with volume as $F^{*}\sim K_{3}V^{\frac{1}{3}}$. For the uniaxial cholesteric we know^8^ that the dominating part of the energy is given by the anchoring term, therefore $F_{u}\sim\gamma\omega V^{2/3}$. Comparing the two energies we get the transition at $V^{*}\sim{(\frac{K_{3}}{\gamma\omega})}^{3}$. This approach misses to predict the correct transition volume, for it being identical to the homogenous to bipolar scaling estimate.

**Variational theory.** At first, we introduce the oblate spheroidal coordinates ($\sigma,\tau,u)$^9^:

$x=\beta\sigma\tau$;

$$y=\beta\sqrt{1+\sigma^{2}}\sqrt{1-\tau^{2}}\cos u;$$

$$z=\beta\sqrt{1+\sigma^{2}}\sqrt{1-\tau^{2}}\sin u.$$

where $\beta\geq0$is a parameter. We prescribe a nematic field, in the basis given by the previous coordinates, as follows:

$$n=\cos\left( q\beta\sigma+u \right)e_{u}-\sin\left( q\beta\sigma+u \right)e_{\sigma}$$

The director field $n$ forms a helix, twisting around the coordinate curve of $\sigma$, with $q$ being the wave number, that is with a pitch equal to $p :=\frac{2\pi}{q}$.

We consider for the shape of the droplet an ellipsoid of aspect ratio $\alpha$, whose axis of symmetry is the $z$-axis in our coordinate system, although for homogenous and bipolar droplets a spindle-like shape would be more accurate. The nematic field and the ellipsoid have two different axes of symmetry, x-axis for the director field and z-axis for the ellipsoid.

To reduce the number of variables, the dependence of $F$ on the volume is made explicit by rescaling $qV^{1/3}=:\tilde{q}$ and $\beta V^{-1/3} =:\tilde{\beta},$ therefore, the integrals can be rewritten independent of volume:

$$F=V^{\frac{1}{3}}\int{[ K}_{1}\left( \nabla\cdot n \right)^{2}+K_{2}\left( n\cdot\nabla\times n \right)^{2}+K_{3}\left( n\times\nabla\times n \right)^{2}] dV+\gamma V^{\frac{2}{3}}\int_{S} 1+\omega\left( n\cdot t \right)^{2} dS-2K_{2}q_{\infty} V^{\frac{2}{3}}\int(n\cdot\nabla\times n) dV+K_{2}q_{\infty}^{2}V.$$

For each value of the parameters $\alpha,\beta$ and $q,$ the integrals were computed numerically with a precision $\Delta x=0.002$ and with a span in the parameters $\Delta\alpha=0.1, \Delta\tilde{\beta}=0.05$ and $\Delta\tilde{q}=0.02$, The free energy was minimized for 200 values of the volume, logarithmically spaced between $10$ and ${10}^{7}$. The various constants were set to the values found in^8^ while the elastic constants were adapted to better fit the data:

$$\gamma=0.6\cdot{10}^{-6}N/m, K_{2}=0.3\cdot{10}^{-5}\mu N,$$

$$\omega=2, K_{1}=K_{3}=0.85\cdot{10}^{-5}\mu N,$$

$$p_{\infty}=15\mu m\Rightarrow q_{\infty}=0.42 \mu m^{-1}.$$

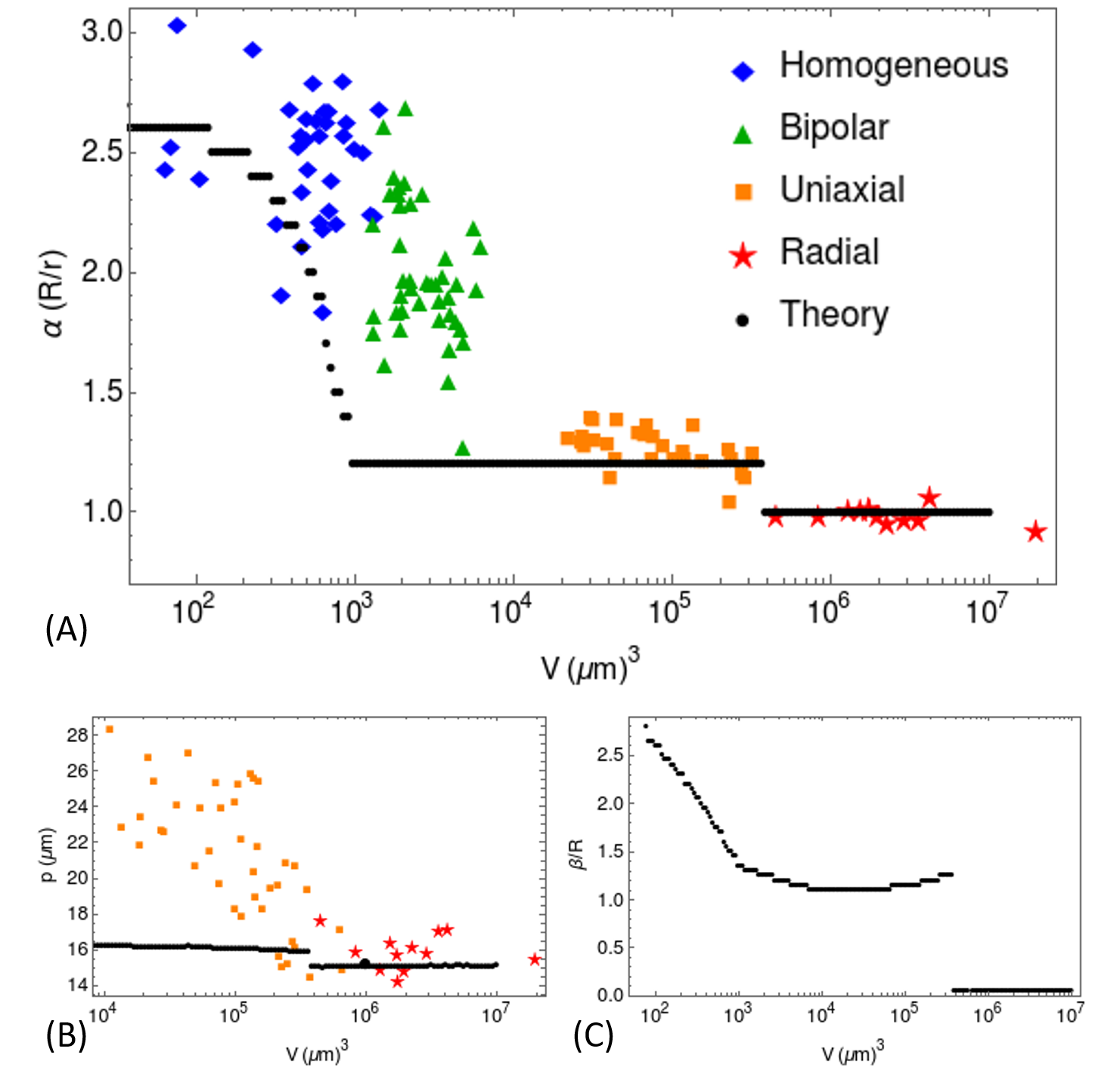


**Figure S7 Results of the minimization of the free energy with experimental data.** A) Aspect ratio as a function of the volume. B) Pitch (p) as a function of the volume, C) $\beta/R$ ratio as a function of the volume.

The minimization reproduces correctly the four phases (Fig.S7). However, the transition volume to the uniaxial cholesteric is overestimated by a factor 10 (Fig. S7A). More importantly, the decreasing behavior of the pitch with volume is not yet captured by the model (Fig. S7B). A possible reason could be given by a difference in concentration in droplets, which are in different growth stages for different volumes. Alternatively, a change of rod length distribution inside the droplet could be taking place in time (i.e., at increasing volumes), which may affect macroscopic quantities. Such effects are evidently beyond the reach of the present model. This problem remains still open, and further analysis will be carried out in the future.

In order to tackle this issue, a modified model was considered, where $p_{\infty}$ was assumed to vary linearly with$\log_{10} V$, in line with experiments. The coefficients of such linear behavior were optimized in order to reproduce the experimental values of the pitch, obtaining $p_{\infty}=(46-5\log_{10} V)\mu m$. Under this assumption, the results shown in Figure S8 were obtained, in very close agreements with experiments.


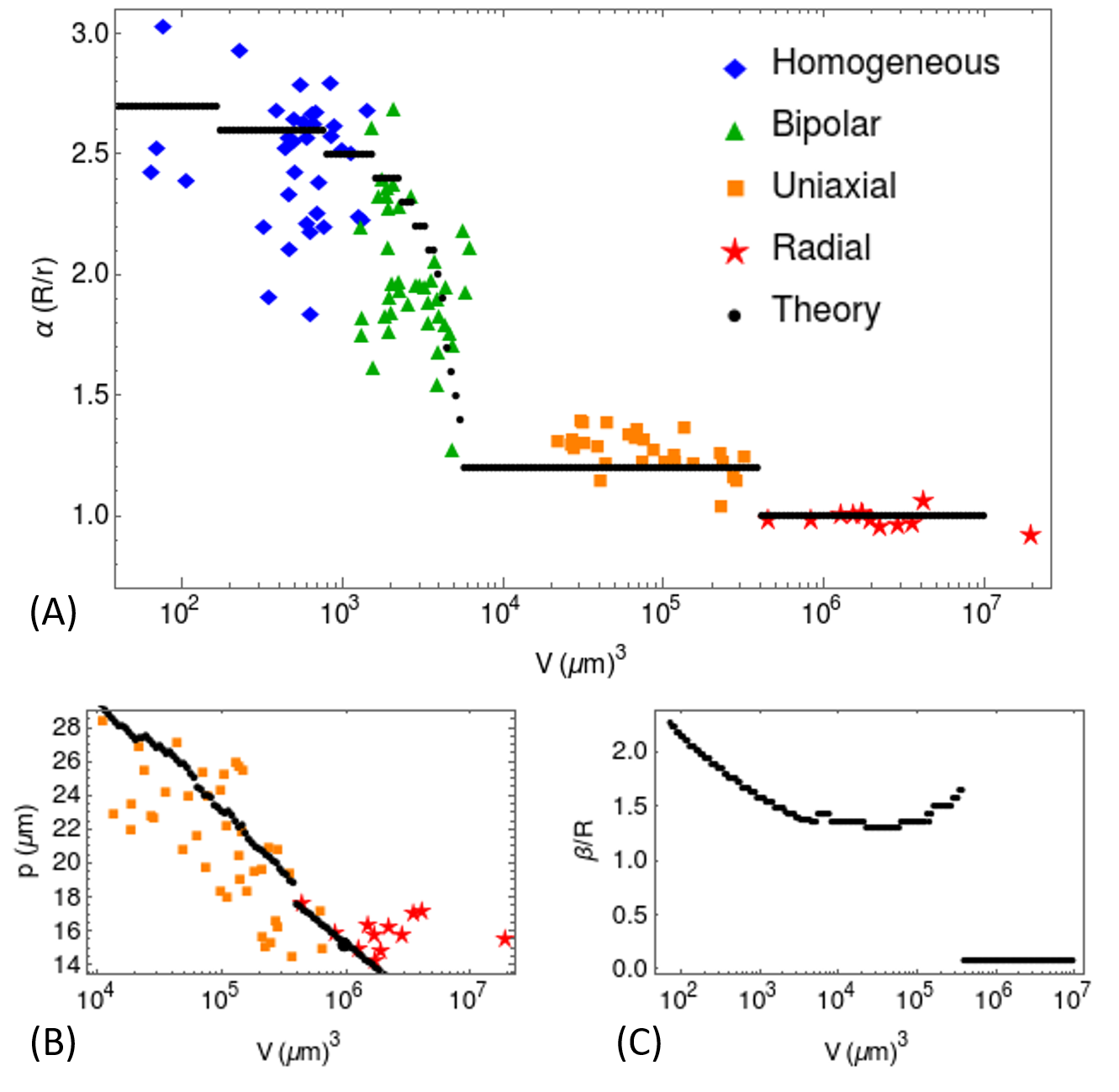


**Figure S8 Results of the minimization of the free energy, and comparison with experimental data.** A) Aspect ratio as a function of the volume. B) Pitch as a function of the volume, C) $\beta/R$ ratio as a function of the volume.

**LC Polscope rendering through Jones matrices** Using the Jones calculus ^10^ with 2 × 2 matrices, we can reproduce the polarized microscopy. The volume of a droplet is divided into cubic elements, where the nematic field is assumed constant. The Jones matrix for each element is calculated using the model shown in the variational theory part and utilizing the parameters of the minimization. Paring the previous matrices with the Jones matrices of two variables retarders^11^ and a circular polarized light, we are able to reproduce the universal compensator device, as explained in ^12^, and therefore map with colors the direction of the measured director field.

**Bibliography**

1. Bagnani, M., Nyström, G., De Michele, C. & Mezzenga, R. Amyloid Fibrils Length Controls Shape and Structure of Nematic and Cholesteric Tactoids. *ACS Nano* acsnano.8b07557 (2018). doi:10.1021/acsnano.8b07557

2. Usov, I. & Mezzenga, R. FiberApp: An open-source software for tracking and analyzing polymers, filaments, biomacromolecules, and fibrous objects. *Macromolecules* **48,** 1269–1280 (2015).

3. Seč, D., Porenta, T., Ravnik, M. & Žumer, S. Geometrical frustration of chiral ordering in cholesteric droplets. *Soft Matter* **8,** 11982 (2012).

4. Leforestier, A. & Livolant, F. Supramolecular ordering of DNA in the cholesteric liquid crystalline phase: an ultrastructural study. *Biophys. J.* **65,** 56–72 (1993).

5. Donald, A. M., Viney, C. & Ritter, A. P. The parabolic focal conic texture in a lyotropic liquid-crystalline polymer. *Liq. Cryst.* **1,** 287–300 (1986).

6. Roman, M. & Gray, D. G. Parabolic focal conics in self-assembled solid films of cellulose nanocrystals. *Langmuir* **21,** 5555–5561 (2005).

7. Virga, E. G. *Variational Theories for Liquid Crystals*. (1995). doi:10.1007/978-1-4899-2867-2

8. Nyström, G., Arcari, M. & Mezzenga, R. Confinement-induced liquid crystalline transitions in amyloid fibril cholesteric tactoids. *Nat. Nanotechnol.* **13,** 330–336 (2018).

9. Morse, P. & Feshbach, H. *Methods of Theoretical Physics, Part I*. (McGraw-Hill, 1953).

10. Lien, A. Extended Jones matrix representation for the twisted nematic liquid‐crystal display at oblique incidence. *Appl. Phys. Lett.* **57,** 2767–2769 (1990).

11. Gerrard, A. (Anthony) & Burch, J. M. (James M. . *Introduction to matrix methods in optics*. (Dover, 1994).

12. Shribak, M. & Oldenbourg, R. Techniques for fast and sensitive measurements of two-dimensional birefringence distributions. *Appl. Opt.* **42,** 3009 (2003).
